# Proteomic profiling of glucocorticoid-exposed myogenic cells: Time series assessment of protein translocation and transcription of inactive mRNAs

**DOI:** 10.1186/1477-5956-7-26

**Published:** 2009-07-30

**Authors:** Erica KM Reeves, Heather Gordish-Dressman, Eric P Hoffman, Yetrib Hathout

**Affiliations:** 1 Research Center for Genetic Medicine, Children's National Medical Center, 111 Michigan Ave NW, Washington DC. 20010, USA

## Abstract

**Background:**

Prednisone, one of the most highly prescribed drugs, has well characterized effects on gene transcription mediated by the glucocorticoid receptor. These effects are typically occurring on the scale of hours. Prednisone also has a number of non-transcriptional effects (occurring on minutes scale) on protein signaling, yet these are less well studied. We sought to expand the understanding of acute effects of prednisone action on cell signaling using a combination of SILAC strategy and subcellular fractionations from C_2_C_12 _myotubes.

**Results:**

*De novo *translation of proteins was inhibited in both SILAC labeled and unlabeled C_2_C_12 _myotubes. Unlabeled cells were exposed to prednisone while SILAC labeled cells remained untreated. After 0, 5, 15, and 30 minutes of prednisone exposure, labeled and unlabeled cells were mixed at 1:1 ratios and fractionated into cytosolic and nuclear fractions. A total of 534 proteins in the cytosol and 626 proteins in the nucleus were identified and quantitated, using 3 or more peptides per protein with peptide based probability ≤ 0.001. We identified significant increases (1.7- to 3.1- fold) in cytoplasmic abundance of 11 ribosomal proteins within 5 minutes of exposure, all of which returned to baseline by 30 min. We hypothesized that these drug-induced acute changes in the subcellular localization of the cell's protein translational machinery could lead to altered translation of quiescent RNAs. To test this, *de novo *protein synthesis was assayed after 15 minutes of drug exposure. Quantitative fluorography identified 16 2D gel spots showing rapid changes in translation; five of these were identified by MS/MS (pyruvate kinase, annexin A6 isoform A and isoform B, nasopharyngeal epithelium specific protein 1, and isoform 2 of Replication factor C subunit 1), and all showed the 5' terminal oligopyrimidine motifs associated with mRNA sequestration to and from inactive mRNA pools.

**Conclusion:**

We describe novel approaches of subcellular proteomic profiling and assessment of acute changes on a minute-based time scale. These data expand the current knowledge of acute, non-transcriptional activities of glucocorticoids, including changes in protein subcellular localization, altered translation of quiescent RNA pools, and PKC-mediated cytoskeleton remodeling.

## Background

The synthetic glucocorticoid prednisone is one of the most widely prescribed drugs worldwide due to its potent immunosuppressive and anti-inflammatory effects [1]. Prednisone treatment remains the standard of care for treatment of a variety of disorders including asthma, muscular dystrophy, autoimmune disorders, and arthritis. Other newer and more potent or targeted immunosuppressants have been tried in many of these disorders, but they have been less efficacious than prednisone. However, chronic use of prednisone is associated with significant negative side effects. A greater understanding of prednisone's molecular actions could help optimize treatments that maximize efficacy while minimizing side effects.

The best characterized effect of steroids is their ability to bind soluble steroid hormone receptors, and then move to the nucleus where the receptor complex directly binds to promoter elements and mediates gene transcription. In the case of glucocorticoids, there are well studied glucocorticoid response elements in target gene promoters that are responsible for binding the ligand/receptor complex, with downstream modulation of gene transcription [2,3]. Synthetic glucocorticoids have been shown in multiple tissues to decrease membrane fluidity by altering the cholesterol/phospholipids ratio [4-6]. While downstream effects of glucocorticoids on gene transcription and regulation have been well characterized, their non-transcriptional signaling response is poorly understood. Prednisone has been shown to acutely activate protein kinase C (PKC) dependent mitogen activated protein kinase pathway via putative G-protein coupled receptors in immune, brain, and lung cells [7-10]. Additionally, glucocorticoids have been reported to translocate annexin A1 and 5-lipoxygenase and S100A11 to the cytoskeleton in response to glucocorticoid exposure [8,11,12].

We have reported the transcriptional effects of glucocorticoids in muscle cells using *in vivo *time series data [13]. Methods for profiling proteins are beginning to emerge, and we envisioned that generating proteomic profiling time series data on the minute time scale, integrated with mRNA changes on the minutes time scale, would enable a systems biology understanding of the mechanisms of action of prednisone. Subcellular proteomic profiling to gauge protein translocation on the minute time scale would add important information regarding the effects of prednisone on cell signaling.

To date, protein translocation studies have involved *in vivo *generation of fluorescent analogs of a target protein followed by its microinjection into live cells [14-16]. The discovery of GFP (and its variants) as an expression reporter in 1997 facilitated the study of protein trafficking [17]. However, this method has its limitations since only a few proteins can be studied at any given time, and there have been no reports in vertebrate cells of subcellular protein translocation on a more global scale. One form of protein translocation is protein secretion to the extracellular milieu (secretome). Stable isotope labeling by amino acid in cell culture (SILAC) has been successfully applied to study the secretome in a pancreatic cancer cell line [18] and human primary retinal pigment epithelial cells [19].

SILAC strategy has been also used successfully to generate a number of protein profiles, measure kinetics of protein turnover, describe protein-protein interactions, as well as quantitative phosphoproteomics [20-22]. SILAC is one of the most accurate methods for subcellular proteome profiling because labeled and unlabeled cells can be mixed before subcellular fractionation and protein extraction, thus minimizing variation due to experimental handling.

Here, we implemented SILAC strategy to expand our knowledge about intracellular protein translocation in response to pharmacological doses of prednisone. We used an acute time series experiment (0, 5, 15, and 30 minutes) to identify a series of rapid effects of prednisone on protein abundance and subcellular location. These changes were validated by documenting changes in translation of quiescent RNA pools, and cytoplasmic to nuclear translocation of annexin A2. These data provide a potential mechanism underlying the previously reported inhibitory effect of prednisone on cell proliferation.

## Results

### SILAC and subcellular fractionation defines the protein translocation in response to prednisone

**Figure 1:**
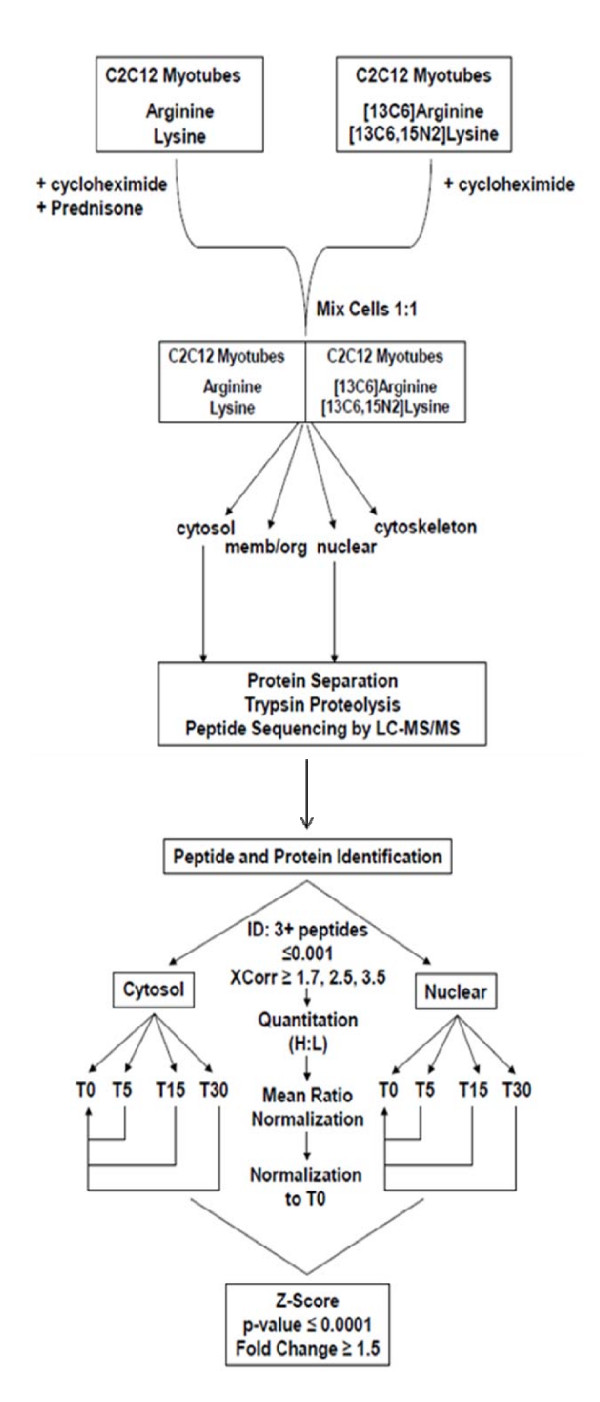
**Methodology for subcellular proteomic assessment in glucocorticoid-exposed myogenic cells**.

We devised a strategy and experimental method to profile protein translocation in response to prednisone exposure *in vitro *(Figure 1). The experimental model used was cultured myogenic (muscle) cells (C_2_C_12 _myotubes) after exposure to a pharmacological dose of prednisone for 0, 5, 15, and 30 minutes. To specifically study protein translocation and not *de novo *changes in protein synthesis, *de novo *protein synthesis was inhibited with cycloheximide throughout all experiments. The glucocorticoid receptor bound to a ligand (i.e. cortisol, prednisone) is well known to modulate transcriptional activity of target genes, and thus secondarily influence protein abundance and localization. However, both the use of cycloheximide to inhibit translation and short time frames largely rule out *de novo *transcription as a significant factor in our studies. Most transcription-mediated protein changes occur on the order of hours, while we used minutes of prednisone exposure [13]. Thus, our experimental design should ensure that any changes in protein abundances in our subcellular fraction are not due to alterations in translation due to *de novo *mRNA or protein production known to occur in response to prednisone.

Pairs of steroid-exposed and non-exposed cells from each time point were mixed at 1:1 ratio (w/w), fractionated into cytosolic and nuclear fractions using differential detergent fractionation then processed for proteome profiling as described in the methods section [23]. The number of proteins identified and quantified for each time point and fraction ranged from 316 to 404 for the cytosolic fraction and 318 to 483 for the nuclear fraction. We found small variances for the ratios of multiple peptides mapping to the same parent protein, indicating the accuracy of SILAC strategy. The percentage of overlapping proteins identified across all four time points ranged from 55–71% and 31–47% in the cytosolic and nuclear fraction respectively (see Additional file 1). The overall identification percentage rate was 41.8% for the cytosolic fraction and 23.8% for the nuclear fraction. The nuclear 5 min time point had a higher number of proteins quantified compared to the other time points in both fractions, exemplifying the issue of variability in performing reproducible independent subcellular fractionation.

### Prednisone drives acute changes in the cytoskeleton

**Figure 2:**
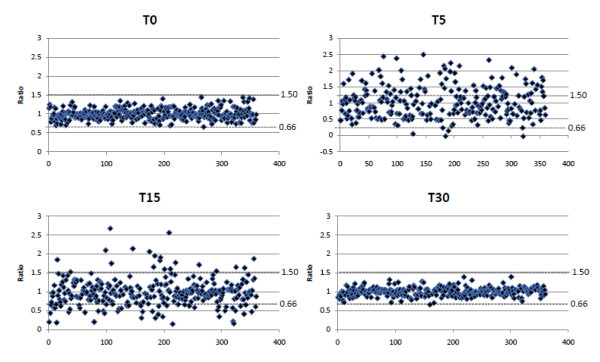
**Significant alterations in protein localization within 15 minutes of prednisone Exposure**. The overall ratio distributions for cytosolic proteins identified across all time points is shown. Protein ratios outside the range of 0.66 and 1.5 were considered significant. Results are derived from a single experiment for each time point. Each sample was comprised of SILAC labeled and unlabeled proteins.

We first analyzed the distributions of all protein ratios in each fraction and time point. Figure 2 show the overall ratio distribution of proteins in the cytosolic and nuclear fractions at different time points following exposure to prednisone. The distributions of ratios for T0 (control) were quite narrow (T0 cytosol: δ = 0.21 and T0 nuclear: δ = 0.22). Effects of the drug on protein ratios were statistically significant within 5 and 15 min of prednisone exposure, with broadening of distributions relative to T0 (cytosol T5: δ = 0.50 (p-value < 0.0001; T15 δ = 0.41 (p-value < 0.001) respectively and δ = 0.32 (p-value = 0.003) and δ = 0.44 (p-value < 0.001) in the nuclear fraction. The distributions at time 30 minutes narrowed considerably, becoming more narrow compared to T0 (cytosol T30 δ = 0.12 (p-value = 0.01); nuclear T30 δ = 0.14 (p-value = 0.001)), suggesting that most protein localizations and/or quantities were changed soon after prednisone application, and that these protein changes largely returned to baseline levels by 30 minutes.

We limited analyses to those proteins identified across all time points for a specific fraction, and those proteins with a set ratio and p-value cutoff. Since T0 ratios typically ranged between 0.66 and 1.5, ratios outside this window were considered significant. After filtering, the number of differentially altered proteins was reduced from 223 proteins to 53 proteins in the cytosolic fraction, and from 149 to 16 proteins in the nuclear fraction (see Additional file 2). Many of the most significant temporal changes were seen in cytoskeleton-related proteins (Table 1). In addition to these proteins listed in the table, there was a significant decrease of myosin heavy chain 8, tropomyosin 1 and troponin C type 1 in either the cytosol or nucleus for the first 15 minutes of exposure to prednisone. While other cytoskeleton and cytoskeleton-associated proteins such as alpha actinin 4, glutaredoxin 3, myoferlin, talin-1 and vimentin were significantly increased in either the cytosol or nucleus for the first 15 min following prednisone exposure. By 30 min, these proteins (with the exception of tropomyosin 4) had returned to basal levels. This suggests an acute cytoskeleton rearrangement in prednisone-exposed cells versus unexposed cells.

**Table 1:** Top proteins significantly altered in response to prednisone

	**Fold Change Up-regulated**			
**Accession**	**Molecules**	**FC**	**SD**	**# Pep ID**
IPI00421223.2	Tropomyosin 4	5.03	0.10	10
IPI00229509.1	Plectin-1	4.07	0.13	14
IPI00229534.4	Myristoylated alanine-rich C-protein	4.00	0.03	7
IPI00131138.9	Filamin A	3.37	0.07	32
IPI00223253.1	Heterogeneous nuclear ribonucleoprotein K	3.34	0.12	9
IPI00380436.1	Alpha-actinin 1	3.10	0.12	4
IPI00113241.6	Ribosomal protein S19	2.67	0.13	9
IPI00124829.4	Actin-related protein 2/3 complex subunit 3	2.56	0.28	5
IPI00111831.1	Nascent polypeptide-associated complex subunit alpha, muscle-specific form	2.11	0.13	51
IPI00126115.1	Sideroflexin-3	2.11	0.05	6

	**Fold Change Down-regulated**			
**Accession**	**Molecules**	**FC**	**SD**	**# Pep ID**

IPI00284119.6	Troponin C, skeletal muscle	-4.35	0.10	13
IPI00312700.3	Myosin light chain 1	-2.78	0.06	11
IPI00228548.5	Enolase 3	-2.77	0.32	28
IPI00225275.4	Glycogen phosphorylase, muscle form	-2.76	0.10	23
IPI00319994.5	L-lactate dehydrogenase A chain	-2.60	0.24	24
IPI00223757.3	Aldose reductase	-2.53	0.28	12
IPI00121788.1	Peroxiredoxin-1	-2.45	0.16	12
IPI00221402.6	Fructose-bisphosphate aldolase A	-2.30	0.18	11
IPI00123319.1	Tropomyosin beta chain	-2.27	0.23	25
IPI00110990.1	Dual specificity protein phosphatase 3	-2.27	0.10	11
IPI00119667.1	Elongation factor 1-alpha 2	-2.24	0.10	8

### Prednisone rapidly alters subcellular localization of mRNA translational members

**Figure 3:**
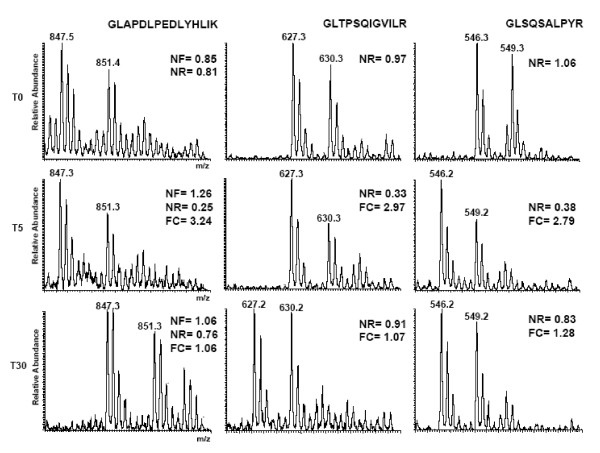
**RPS13 increases in the cytosol in response to prednisone**. Quantitation of the zoom scans for three unique peptides identified in the cytosolic fraction for RPS13. The mean ratio for RPS13 was multiplied by each timepoint's normalization factor (NF), which was calculated to adjust the overall protein population mean ratio to 1.0. Peptides were normalized to their corresponding baseline peptide at T0, resulting in a normalized peptide ratio (NR). The NR was then converted to a fold change (FC). Results showed an increase in RPS13 levels detectable through T15 (15 minutes) post-exposure. Quantitation was performed on the intercalating zoom scans for the corresponding MS2 scan by taking the ratio of the monoisotopic peak of the heavy (labeled) peak divided by the monoisotopic peak of the light (unlabeled, prednisone-exposed) peak.

**Figure 4:**
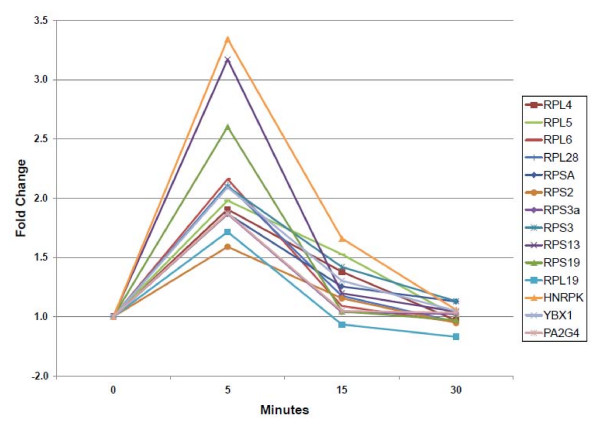
**Temporal profiling of transcriptional/translational proteins showing acute translocation into the cytosol**. Shown are relative ratios of ribosomal proteins and transcriptional regulators with significant changes in the cytosol following exposure to prednisone. All proteins reached their maximum levels at five minutes and returned to baseline levels by 30 minutes.

A list of proteins showing significant changes in either the cytosol or nuclear fractions (see Additional file 2) were imported into GO-Getter  to obtain gene ontology analysis. The largest percentage (21.7%) of proteins from this list was categorized as ribosomal proteins (data not shown). Eleven ribosomal proteins were found to be significantly increased in the cytosolic fraction at 5 min following prednisone exposure (Table 2; Figures 3 and 4). Increased ribosomal proteins included subunits that form the 40S and 60S ribosomal complex. All of these proteins shared a common transient increase at 5 min post-prednisone treatment with a return to basal levels by 30 min post-treatment. In addition to the ribosomal proteins, other proteins involved in transcription/translation also had altered subcellular localization, including elongation factor 1 alpha 1 (EEF1A1), elongation factor 1-alpha 2 (EIF1A2)) and the transcriptional regulators heterogeneous nuclear ribonucleoprotein K (HNRPK), y-box binding protein 1 (YBX1) and proliferation-associated 2G4 (PA2G4) (Figure 4).

**Table 2:** Transient increase in ribosomal proteins in the cytosol

		T5		T15		T30	
IPI Accession No.	Protein	FC	p-value	FC	p-value	FC	p-value
IPI00111272.1	PREDICTED: similar to 40S ribosomal protein SA	1.87	0.00000	1.26	0.04298	1.13	0.226600
IPI00111412.2	60S ribosomal protein L4	1.91	0.00000	1.38	0.00169	0.97	0.510430
IPI00113241.6	40S ribosomal protein S19	2.60	0.00000	1.04	0.32051	0.97	0.885180
IPI00125901.4	40S ribosomal protein S13	3.17	0.00000	1.20	0.60448	1.04	0.079524
IPI00134599.1	40S ribosomal protein S3	2.10	0.00000	1.42	0.00167	1.13	0.439200
IPI00134607.5	PREDICTED: similar to 40S ribosomal protein S2	1.59	0.00000	1.16	0.09428	0.95	0.476900
IPI00222547.5	60S ribosomal protein L28	2.11	0.00001	1.18	0.00304	0.96	0.816390
IPI00282248.1	PREDICTED: similar to 60S ribosomal protein L6	2.16	0.00000	1.09	0.61050	0.96	0.177500
IPI00308706.3	60S ribosomal protein L5	1.98	0.00000	1.53	0.44499	1.01	0.061444
IPI00331345.4	40S ribosomal protein S3a	1.87	0.00000	1.04	0.00000	1.02	0.889755
IPI00122426.1	60S ribosomal protein L19	1.72	0.00000	0.93	0.65748	0.83	0.321220

**Figure 5:**
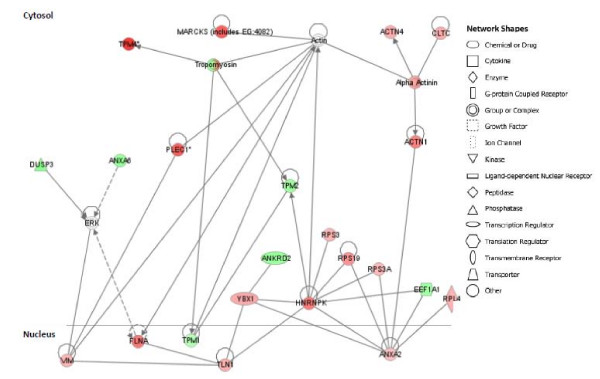
**Network analysis of translocated proteins**. All proteins (n = 69) showing significant translocation in or out of the cytosol or nuclei following prednisone exposure were loaded into Ingenuity Pathway Analysis. This analysis queries published protein-protein or gene-protein interactions between members of the 69 protein set. Proteins were placed in the subcellular fraction in which they were found to change abundance. Red indicates an increase in that fraction, green a decrease. This network includes a sub-network centered on ANXA2 and is connected with ribosomal proteins and other proteins involved in translational machinery. White color indicates proteins mapped to the network that were part of the imported data. Solid line indicates a direct interaction between molecules. Dashed lines indicate an indirect interaction between molecules.

The list of proteins used for gene ontology analysis was also imported into Ingenuity Pathway Analysis (IPA). IPA integrates proteomics profiles with a manually-curated database of existing literature. This tool enables the interpretation of data in the context of biological significance. IPA allows statistical ranking of both "canonical pathways" (well-established biochemical pathways) and "networks" (protein-protein and gene-protein interactions suggested by the literature). Top ranked networks are defined as those showing the greatest number of connections within a 35 protein 'space'. Analysis of the 69 proteins showing significant subcellular translocation in response to prednisone resulted in a single highly ranked network that contained 25 of the 69 proteins imported. This network contained multiple sub-networks centered on proteins including actin, myosin, HNRPK and annexin A2 (ANXA2) (Figure 5). Many of the proteins mentioned above mapped to this network, further supporting the hypothesis that protein synthesis and cytoskeletal remodeling are significantly affected by prednisone treatment.

**Figure 6:**
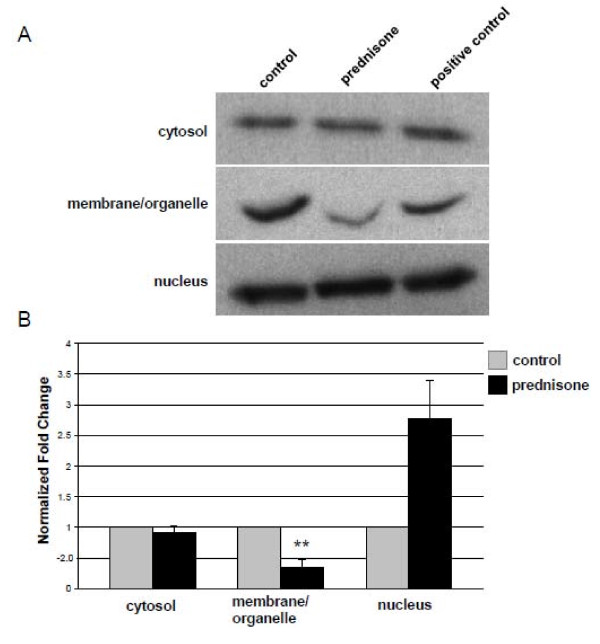
**ANXA2 translocates from the membrane/organelle fraction to the nucleus in response to prednisone**. Shown is immunoblot analysis of C2C12 subcellular fractions probed for ANXA2 (A), and quantitation of experimental triplicates densitometry (B). ANXA2 was not detected in the cytoskeleton fraction (data not shown). ANXA2 migrated from the membrane/organelle fraction to the nucleus within 15 minutes of prednisone exposure (**= p ≤ 0.05). Data in Panel B are mean ± SE.

While SILAC is one of the most accurate protein quantitative methods, we used immunoblotting as a secondary method to validate a subset of our data. ANXA2 was chosen due to its central position in the network shown in Figure 5, as well as the availability of antibody reagents. The cytosolic, nuclear, membrane/organelle, and cytoskeletal fractions pre- and post-exposure with glucocorticoids were probed for ANXA2 (Figure 6). Consistent with the mass spectrometry data, we found a 2.7-fold increase of ANXA2 in the nucleus, however this did not reach statistical significance by experimental triplicates due to high variance of the immunoblot results (p = 0.06). More importantly, immunoblot analysis of the membrane/organelle fraction showed a significant 2.8-fold decrease of ANXA2 (p = 0.02) (Figure 6). These data demonstrate that prednisone induces rapid intracellular migration of ANXA2 from the membrane/organelle compartment to the nuclear compartment and also validate the mass spectrometry quantitation findings.

### Acute protein stability and translational response to prednisone

ANXA2, HNRPK and the majority of proteins networked with these two proteins are involved in the regulation of translationally-inactive messenger ribonucleic acids (mRNAs). We hypothesized that perturbations in translational machinery would lead to downstream changes in the translation of inactive mRNA pools. If true, we would expect to see acute changes in the translation of specific mRNAs in response to prednisone over this time frame, possibly involving traffic of mRNAs to or from quiescent RNA pools.

To test if there was a change in pattern of acute mRNA translation, C_2_C_12 _muscle cells were simultaneously exposed to prednisone and ^35^S-Methione/Cysteine for 15 min in the absence of cycloheximide. Transcriptional inhibitors were not used because they have been shown to activate transcription of quiescent pools of mRNAs [24]. Cytosolic proteins were analyzed by 2D gels, with fluorography to detect *de novo *synthesized proteins over the 15 min time frame. All experiments were carried out in biological triplicate, and automated quantitative analyses of autoradiography gels performed by Ludesi.

**Figure 7:**
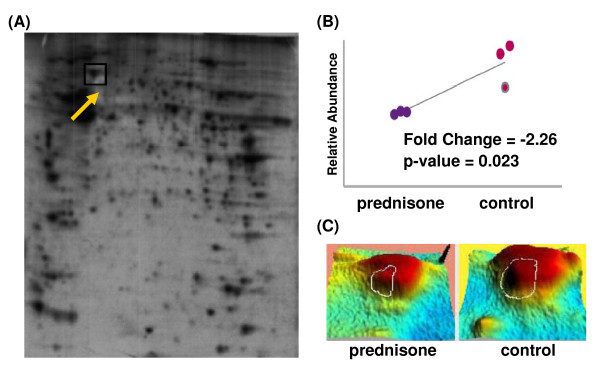
**^35^S-Methionine/Cysteine labeling of *de novo *protein translation identifies reduced ANXA6 isoform A protein translation patterns**. One of the 5 proteins showing altered translation patterns was Annexin A6 (ANXA6) isoform A which decreased 2.23 in response to prednisone. (A) Original prednisone-exposed autoradiography gel. Arrow points to location of ANXA6.A. (B) Normalized means of relative protein abundance for triplicate experiments. (C) Enlarged view of the resolved ANXA6.A spot of the 3D rendering of the 2D montage.

A total of 832 different spots were detected between the control and prednisone gels. Analysis was limited to spots identified in all gels in both groups (n = 274) or those identified in all three gels from a single group (exposed n = 83; unexposed n = 81) (n = 438 non-redundant spots). Fifteen spots had a fold change ≥ 1.5 and p value ≤ 0.05 in the exposed group when compared to unexposed controls (Figure 7). These spots were selected for peptide mass fingerprint identification using Coomassie stained gels. Eight of the fifteen proteins were abundant enough to be detected on the less sensitive Coomassie stained gel and could be confidently mapped and excised. Of these eight proteins, five proteins showed adequate MS/MS results for unambiguous identification (Table 3).

**Table 3:** Altered *de novo *translation patterns in response to prednisone

Fold Change	p-value	Protein	Accession #	C.I	#TOP motifs
1.79	0.027	Pyruvate kinase isozyme M2	IPI00407130	98.7	6
-2.26	0.023	Annexin A6 isoform A	IPI00649152	100	6
1.68	0.017	Annexin A6 isoform B	IPI00310240	100	6
-3.55	0.039	Nasopharyngeal epithelium specific protein 1	IPI00474207	100	6
Control Only	0.046	Isoform 2 of Replication factor C subunit 1	IPI00378168	99.8	4

The 5' untranslated region (UTR) of the genes corresponding to each of the proteins was analyzed for RNA regulatory motifs using the publicly available web server RegRNA . Each of the five proteins contained multiple 5' terminal oligopyrimidine tracts (TOP) (Table 3). Both nasopharyngeal epithelium specific protein 1 (NESG1) and isoform 2 of replication factor C subunit 1(RFC1) also contained Gamma interferon activated inhibitor of Ceruloplasmin mRNA translation (GAIT) elements.

## Discussion

We defined acute (minutes of treatment exposure time scale) protein changes in subcellular fractions in response to prednisone with time series data, enabling some delineation of cause/effect of observed changes in protein subcellular localization. Our experimental design involved a SILAC strategy to monitor temporal protein translocation in prednisone-exposed C_2_C_12 _cells (Figure 1). We used stringent criteria for protein identification and quantitation to reduce the number of false positive identifications and to provide an assessment of variance of peptide ratios between time points.

The percentage of proteins identified across all time points (e.g. 0, 5, 15, and 30 min exposure) is low when compared to the total number of identified proteins (41.8% in the cytosol and 23.8% in the nucleus) and this is due to the poor reproducibility when dealing with multiple subcellular fractionations (see Additional file 1). However, these percentages are higher than previously reported values. Currently, there are only a handful of published papers using a SILAC-based LC-MS/MS in temporal studies. Additionally, not all of these published papers address protein identification rates across samples and fractions. A recent paper published from our group (focused on the endoplasmic stress response) found 19.7% of total identified proteins overlapping across four time points [25]. Another study (focused on concurrent temporal protein expression patterns during B-cell differentiation) found 23.4% overlapping proteins across five time points [26]. Our higher percentages could be attributed to more stringent filtering criteria.

Significantly altered proteins between what and what were imported into Ingenuity for network analysis. Many of the molecules in the highest ranked network were cytoskeleton-related proteins (Table 1; Figure 5). These proteins, in addition to the numerous other cytoskeleton proteins found to significantly change localization in response to prednisone, suggest an acute cytoskeleton remodeling of muscle cells in response to prednisone exposure. Glucocorticoid-induced actin remodeling has not been demonstrated in muscle but has been found in other tissues. In keratinocytes, glucocorticoids induced rapid actin assembly in endometrial cells [27,28]. A dose-dependent decrease in the G/total actin ratio was seen with a concurrent increase in F-actin in as early as 15 minutes of exposure.

Numerous studies have implicated the activation of PKC and PKC-mediated cytoskeleton remodeling in response to glucocorticoids in non-muscle tissues [10,29-31]. In the present study, several PKC binding proteins showed alterations in protein localization, including EEF1A, EEF1A2, myristoylated alanine-rich C-protein (MARCKS) and glutaredoxin-3 (GLRX3) in or out of the cytosol. GLRX3 (also known as PICOT) was shown to bind calcium-independent PKCθ. In unstimulated T-cells, GLRX3 is distinctly localized to the cytoplasm. PKCθ stimulation results in the co-translocation of PKCθ and GLRX3 to a submembrane location [32]. In cardiac muscle, GLRX3 interacts with muscle LIM protein located at cardiac Z-disks, where it interacts with other Z-disk proteins such as alpha-actinin, zyxin, and T-cap [33]. GLRX3 competitively displaces calcineurin and disrupts dephosphorylation of NFAT and subsequent downstream signaling. GLRX3 may also bind specific PKC isozymes inhibiting downstream MAPK activity. The author suggests that this scaffolding domain functions as an inhibitor of the hypertrophic signal transduction. This model could be applied to the atrophic response of skeletal muscle to glucocorticoids.

Another PKC substrate, MARCKS, decreased in the cytosol. MARCKS is an actin filament cross-linking protein normally localized to focal adhesions at the plasma membrane. Phosphorylation by PKC inhibits its association with actin at the plasma membrane and results in increased cytosolic localization [34]. Glucocorticoid treatment rapidly increased MARCKS 4.2-fold in the cytosol. In muscle, PKC-dependent translocation of MARCKS correlates with integrin signaling and actin cytoskeleton remodeling [35]. MARCKS transcription is downregulated by glucocorticoids [13]. Other integrin signaling proteins, including actin related protein complex subunit 3 and alpha actinin 1 and 4 and numerous cytoskeleton-related proteins, also significantly altered localization further suggesting an acute cytoskeletal remodeling occurring. Taken together, our data suggests there is a potential non-transcriptional PKC-mediated cytoskeletal remodeling occurring within minutes of prednisone exposure. However, further experiments are required to validate this result and delineate which molecular mechanisms are responsible.

In addition to the cytoskeletal and cytoskeleton-bound proteins, there were significant changes seen in proteins involved in protein synthesis. Multiple ribosomal proteins and transcriptional regulators increased in the cytosolic fraction upon glucocorticoid treatment (Table 2; Figures 3 and 4). All of these proteins shared a common temporal pattern, with the greatest increase in the cytosol seen within 5 minutes of prednisone exposure and returning to basal levels by 30 minutes (Figures 3 and 4). We hypothesize that the ribosomal proteins are translocating into the cytosolic fraction from either the cytoskeleton or the membrane/organelle fraction. An alternative interpretation is that these proteins may be stabilized by drug treatment. Unfortunately, antibody reagents are not currently available to test these models. We were able to validate the ANXA2 mass spectrometry findings using immunoblot, and by testing additional fractions showed that the intracellular movement was likely translocating from the membrane/organelle to the nuclear compartment (Figure 6). ANXA2 has been observed in multiple subcellular compartments and is known to traffic between the cytosol and nucleus [36]. Nuclear retention of ANXA2 results in reduced cell proliferation in prostrate cells [37]. Extrapolating from these data, the steroid-induced migration of ANXA2 from the membrane/organelles to the nucleus may be, at least in part, responsible for the reduced C_2_C_12 _proliferation observed in response to glucocorticoids [38]. This is the first report that we are aware of demonstrating glucocorticoid-induced alteration of ANXA2 localization in skeletal muscle.

Glucocorticoids have a receptor-mediated transcriptional response that alters transcriptional profiles. This is the first report demonstrating that ribosomal proteins and transcriptional regulators (i.e. ANXA2, HNRPK, YBX1, etc) have a non-transcriptional mechanism of action. We hypothesized that the observed rapid increase of ribosomal proteins into the cytosol by 5 minutes of steroid treatment could lead to acute changes in mRNA translation patterns, perhaps via sequestering of mRNAs to or from inactive mRNA pools. To test this, we exposed C_2_C_12 _myotubes with ^35^S-Methione/Cysteine and prednisone for 15 minutes to label all *de novo *protein translation products rapidly induced by drug treatment. Fifteen spots showed a fold change ≥ 1.5 and p-value ≤ 0.05 and, of these 15, five were abundant enough to identify by LC-MS/MS (Table 3; Figure 7).

If these five proteins were translated from quiescent RNA pools, then the corresponding 5' UTR of the mRNAs would be expected to contain TOP motifs commonly associated with components of these inactive mRNA pools [39]. All five proteins showing drug-induced acute translation showed multiple TOP motifs in the 5'UTR of their corresponding mRNAs. 6.6% of all mRNAs show ≥ 1 TOP motif(s) [40], and our finding of 5/5 showing multiple TOP motifs was compelling (probability of 1 in 1.2 × 10^-6^). Our data suggests that prednisone is sequestering mRNAs to or from the inactive mRNA pools, leading to a rapid shift in ribosomal proteins to the cytosol. This response appears short-lived, with re-equilibration of the protein translocation pattern within 30 minutes of treatment. However, *de novo *protein translation would be expected to show a more sustained effect.

Translation of TOP mRNAs is dependent upon the growth status of the cell. In growing cells, TOP mRNAs are localized at polysomes and are actively transcribed whereas in quiescent cells, TOP mRNAs are localized at small polysomes and translationally-inactive messenger ribonucleoproteins, where they can be stored for up to a week [41]. Many of the proteins involved in proteins synthesis are represented in the ANXA2-centered sub-network (Figure 5) described above, and are involved in translation of inactive mRNAs. ANXA2 and PA2G4 are both RNA-binding proteins that are present in pre-ribosomal ribonucleoprotein complexes [42,43].

Three of the *de novo*-translated proteins showed an increase in translation, while two showed a significantly decreased rate. The two negatively regulated proteins (NESG1 and RFC1) contain GAIT elements in their 5' UTR. A GAIT element is a cis-acting RNA element involved in selective translational silencing of ceruloplasmin by interferon gamma-activated inhibitor of translation [44]. It has been suggested that the translational silencing of ceruloplasmin and other GAIT-containing transcripts may aid in resolving the local inflammatory response. It is possible that decreases of translation observed in NESG1 and isoform 2 of RFC1 could be attributed to translation silencing via this same mechanism. Overall, it seems that 5'TOP motifs are involved in the acute non-translational response of myotubes to prednisone, with additional motifs controlling whether a shift from or to the quiescent mRNA pools is observed.

## Conclusion

In summary, we describe a systematic approach for monitoring intracellular protein translocation using SILAC and apply this to the acute non-transcriptional response of myogenic cells to prednisone. We found an acute translocation of many cytoskeleton and cytoskeleton-bound proteins indicative of rapid remodeling. Additionally, we discovered the translocation of ribosomal proteins into the cytosol, which was commensurate with *de novo *translation patterns in a subset of mRNAs. These mRNAs were translated within 15 minutes of exposure, and showed features consistent with transport in or out of quiescent pools. The translocation of ribosomal proteins rapidly returned to baseline by 30 minutes, while the *de novo *translation patterns likely have a more persistent drug-induced effect.

## Methods

### Tissue culture methods

Mouse C_2_C_12 _myoblasts were grown in DMEM supplemented with 10% FBS and 1% Penicillin/Streptomycin. Concurrently, equal amounts of mouse C_2_C_12 _cells were incubated in custom-made DMEM medium depleted of Arg and Lys and supplemented with ^13^C_6_-Arg (147.5 ug/mL) and ^13^C_6_, ^15^N_2_-Lys (91.25 ug/mL), as well as 10% FBS and 100 U/mL each of penicillin/streptomycin. The presence of 10% whole serum in the culture medium does not interfere with the incorporation of the exogenous stable isotope labeled amino acids and can be used in place of dialyzed FBS serum [45]. All cells were sub-cultured for five passages to allow full incorporation of isotopes into the cells grown in labeled medium. At five passages, full isotope incorporation was achieved. All cells were serum-starved into differentiation using DMEM supplemented with 2% horse serum and 1% Penicillin/Streptomycin.

### Steroid exposure and subcellular fractionation

On day five of differentiation, both SILAC labeled and non-labeled cells were exposed for 1 hr to 35 μM cycloheximide to arrest translation prior to steroid treatment. Unlabeled myotubes were exposed to 1 μM of Prednisone for 5, 15, or 30 minutes while SILAC labeled cells were unexposed. At each time point steroid-exposed cells were mixed, at 1:1 ratio (w/w), with the non-exposed SILAC labeled cells fractionated using the differential detergent extraction method [23]. Mixing samples prior to fractionation controls for differences in experimental handling. For a control, labeled and unlabeled cells at the 0 minute time point were exposed with cycloheximide but not prednisone. Cytosolic and nuclear fractions were processed for LC-MS/MS analysis.

### Peptide extraction

100 μg of total protein for each fraction and time point was desalted and dry vacuumed to completion, brought up in 30 uL Laemmle buffer containing 50 mM DTT, and incubated for 30 min at room temperature. 1D protein separation was performed using a precast 4–12% SDS-PAGE gel, fixed using methanol:water:acetic acid (45:50:5, vol/vol/vol) for 30 min and stained with Coomassie blue. The fractionated lane was excised into 35 bands. In-gel digestion was performed [46]. Extracted peptides were dissolved in 0.1% TFA.

### LC-MS/MS analysis quantitative and statistical analysis of proteins

All analyses were carried out on a Thermo LTQ Linear ion-trap connected to a nano-LC system. Each sample was injected via an autosampler and loaded onto a C18 trap column (5 μm, 300 μm i.d. × 5 mm), for 6 min at a flow rate of 10 μL/min, 100% A. The sample was subsequently separated by a C18 reverse-phase column (3.5 μm, 100 μm × 15 cm) at a flow rate of 200 nL/min. The mobile phases consisted of water with 0.1% formic acid (A) and 90% acetonitrile with 0.1% formic acid (B). A 100 min linear gradient from 5 to 60% B was typically employed. The LTQ was operated in data-dependent mode in which one cycle of experiments consisted of one full-MS survey and subsequently three sequential pairs of intercalated zoom scans and MS/MS experiments. The targeted ion counts in the ion trap during full-MS, zoom scan, and MS/MS were 30000, 3000, and 10000, respectively. Peptides were fragmented in the linear ion trap using collision-induced dissociation with the collision gas (helium) pressure set at 1.3 milliTorrs and the normalized collision energy value set at 35%. The zoom scan events are of higher resolution higher than and were used to determine the charge state of the ion as well as the ratio of labeled to unlabeled peptide pairs.

### Data base search and quantitative analysis

MS/MS spectra files from each LC run were searched using Sequest against the publicly available mouse EMBL-EBI International Protein Index (IPI) database v3.22 with the following variable parameters: oxidized methionine (15.0 Da) and phosphorylation of serine, threonine, and tyrosine (80.0 Da), and [13C6, 15N2]-lysine (8.0 Da) and [13C6]-arginine (6.0 Da) as variable modifications, fully enzymatic trypsin cleavage specificity allowing for two missed cleavages and 0.8 Da for MS/MS ions. Results were filtered using the following criteria: different peptides, delta CN = 0.1, XCorr vs. Charge State = 1.9, 2.5, and 3.5 (for singly, double, and triply charged peptides respectively), peptide probability = 0.001, number of different peptides = 3. Manual quantitation was performed, where the ratios of at least 3 different peptides were quantitated by dividing the relative abundance of the monoisotopic peak of the heavy peptide by the relative abundance of the monoisotopic peak of the light peptide. Mean and standard deviation of the unexposed/exposed peptide ratios were calculated for each identified protein using all peptides available (n ≥ 3). To adjust for any error in the mixing of labeled and unlabeled cells prior to subcellular fractionation, the average ratio obtained for each protein in a given subcellular fraction and at a given time point was normalized to the average median mean value obtained for that data set. Protein ratios in each of the steroid-exposed sets (T5, T15, and T30) were normalized to the control (T0) by subtracting the control mean for a given protein from the steroid-exposed mean of the same protein. This normalized ratio was used to calculate a z-score and corresponding p-value, indicating if each protein in the steroid-exposed condition was significantly different from the unexposed population. Fold changes for all proteins were also calculated. Results are derived from a single experiment for each time point. Each sample is comprised of SILAC labeled and unlabeled proteins.

### Immunoblot analysis

C_2_C_12 _myoblasts were grown and differentiated under conditions described above. Five days post-differentiated, myotubes were exposed to prednisone for 15 minutes and fractionated using the differential detergent fractionation as described above. Positive controls were 30 ug total protein from NIH/3T3 whole cell lysates. 30 ug of total protein from each subcellular fraction was separated under reducing conditions via one dimensional separation on a 10% SDS-PAGE gel. Proteins were transferred onto a PVDF membrane and blocked in 5% non-fat dried milk in TBS/0.05% Tween 20 (TBST) for 1 hour. Membranes were incubated overnight with an ANXA2 specific antibody; Santa Cruz Biotechnology Inc.). After three washes with TBST, membranes were incubated at room temperature for 1 h using a peroxidase-labeled (HRP) secondary antibody (Santa Cruz) in 5% non-fat dried milk in TBST. After three washes in TBST, bound antibodies were detected by the chemiluminescent method (ECL, Amersham Biosciences). Quantitative analyses were performed using Bio-Rad GS imaging densitometer using QuantOne 4.6.2 software.

### Labeling of acute *de novo *protein translation

C_2_C_12 _myotubes were grown in unlabeled media as described above. On day five post-differentiation, myotubes were exposed to either 250 mCi ^35^S-Methionine/Cysteine or 1 μM Prednisone and 250 mCi ^35^S-Methionine/Cysteine for 15 minutes. Each condition was carried out in triplicate cultures. Cytosolic proteins were isolated as described above. Total cytosolic protein (200 μg) was buffer-exchanged (Tris-HCl, pH 7.4), concentrated, and then solubilized in isoelectric focusing rehydration buffer using a solution consisting of 7 mol/l urea, 2 mol/l thiourea, 1% (wt/vol) ASB-14, 0.5% (vol/vol) Triton X-100, 40 mmol/l Tris base, 30 mmol/l DTT, and 0.5% (vol/vol) ampholytes 3–10. Samples were then loaded onto a 17-cm, pH 3–10 NL Immobilized pH Gradient strip and left under mineral oil overnight. IEF was conducted for a total of 100,000 Vh (volt-hours) at 20°C. Strips were incubated for 15 min in equilibration buffer (6 mol/l urea, 2% [wt/vol] SDS, 20% [vol/vol] glycerol, 0.15 mol/l bis-Tris, and 0.1 mol/l HCl) at 20°C, first with 65 mmol/l DTT and second with 243 mmol/l iodoacetamide. Strips were inserted into 12% SDS-PAGE gels and two-dimensional gels were run according to manufacturer instructions. After protein separation, the gel slabs were fixed in a solution of methanol:water:acetic acid (45:50:5, vol/vol/vol) and then in Amplify Fluorography (Amersham Biosciences) for 30 min. Gels were dried overnight under vacuum seal and stored at -80°C for 25 hrs prior to film development.

### 2D-Gel quantification and analysis

Gel images were analyzed by Ludesi. For spot identification, the same experimental design described above was used but without radioactivity. Non-radioactive gels followed the same procedure with the following exceptions. Cells were exposed to only prednisone for 15 min. After second-dimension separation, gels were fixed (45% dH20/5% acetic acid/50% methanol) followed by three washes for 5 min in deionized water. Staining was performed with Coomassie Blue followed by ample rinsing with deionized water until the desired contrast was obtained.

Spots were excised and transferred to a microcentrifuge tube containing 100 μl of deionized water. Tryptic digestion was performed as described previously [46] and peptides were extracted and then dried by vacuum centrifugation. Dried peptides were subsequently dissolved in 10 μl of 0.1% trifluoroacetic acid and desalted using C_18 _ZipTip micropipette tips following the manufacturer's instructions. Aliquots of peptide solutions were spotted on the matrix-assisted laser desorption/ionization (MALDI) plate. Mass spectrometry (MS) and tandem mass spectrometry (MS/MS) analyses were performed on a 4700 ABI time-of-flight (TOF)-TOF mass spectrometer equipped with a Nd:YAG 200 Hz laser. The instrument was operated with delayed extraction in reflectron positive ion mode. A mixture of standard peptides was used to externally calibrate the instrument. Protein identification was carried out using GPS explorer v3.6 and searched against the IPI database v3.22. Both MS and MS/MS data were used for protein identification. In-gel digestion was performed as described above.

## Abbreviations

ANXA2: Annexin A2; EEF1A1: elongation factor 1 alpha 1; GAIT: Gamma interferon activated inhibitor of Ceruloplasmin mRNA translation; GLRX3: glutaredoxin-3; HNRPK: ribonucleoprotein hnRNP K; IPA: Ingenuity Pathway Analysis; mRNA: messenger ribonucleic acid; NESG1: nasopharyngeal epithelium specific protein 1; NF: normalization factor; PA2G4: proliferation-associated 2G4; PKC: protein kinase C; RFC1: replication factor C subunit 1; SILAC: stable isotope labeling of amino acids; TOP: terminal oligopyrimidine; UTR: untranslated region; YBX1: Y-Box binding protein 1

## Competing interests

The authors declare that they have no competing interests.

## Authors' contributions

ER carried out all proteomic and molecular studies and drafted the majority of the manuscript. HGD helped performed statistical analysis on the proteomic quantitation data. EH made substantial contributions to the study conception and design and critically revised the manuscript for intellectual content. YH contributed to study conception and design, data analysis and interpretation. All authors have read and approved the final manuscript.

## Supplementary Material

Additional file 1 **Protein identifications across time points of prednisone treated myotubes**. The number of overlapping proteins between the time points for the cytosol and nuclear fractions is shown. Click here for file

Additional file 2 **Significantly altered proteins in response to prednisone**. List of cytosolic and nuclear proteins significantly altered in response to prednisone. This list was included for network analysis using Ingenuity Analysis Pathway. Click here for file
