# Supplementary material for: Proteomic profiling of glucocorticoid-exposed myogenic cells: Time series assessment of protein translocation and transcription of inactive mRNAs
Source: Proteome Sci. 2009 Jul 30;7:26. doi: 10.1186/1477-5956-7-26 (PMC2725035; doi:10.1186/1477-5956-7-26)
Supplement: Additional file 1 — Protein identifications across time points of prednisone treated myotubes. The number of overlapping proteins between the time points for the cytosol and nuclear fractions is shown. [file 1477-5956-7-26-S1.doc]

**Additional file 1: Protein identifications across time points of prednisone treated myotubes.** The number of overlapping proteins between the time points for the cytosol and nuclear fractions is shown. The fact that there are 4 time points prevents the overlap between all time points being shown. The missing overlapping values are: cytosol and nuclear T0 and T30 are 16 and 11 respectively. Cytosol and nuclear T5 and T15 are 15 and 35 respectively.

**Additional file 1.**

**
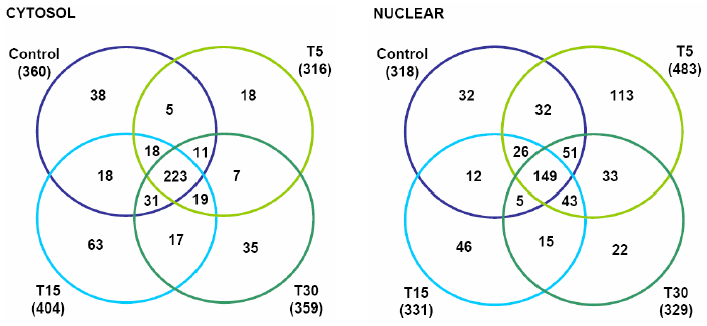
**
